# Chloridotris[tris­(4-fluoro­phen­yl)phosphine]rhodium(I) methanol solvate

**DOI:** 10.1107/S1600536808005485

**Published:** 2008-03-05

**Authors:** Fabio Lorenzini, Brian O. Patrick, Brian R. James

**Affiliations:** a Department of Chemistry, University of British Columbia, 2036 Main Mall, Vancouver, Canada BC V6T 1Z1

## Abstract

In the title compound, [RhCl{P(*p*-FC_6_H_4_)_3_}_3_]·CH_3_OH, the Rh atom adopts a distorted square-planar geometry. Rh, Cl and one P atom lie on a mirror plane, as does the solvent molecule. There are two inter­molecular hydrogen bonds, one between the methanol O atom and an aryl H atom (2.51 Å), and one between the Cl atom and the hydr­oxy H atom of methanol [2.34 (3) Å]. The complex precipitates in trace amounts from a reaction between RhCl(cod)(thp) [cod is 1,5-cyclo­octa­diene and thp is tris­(hydroxy­meth­yl)phos­phine] and P(*p*-FC_6_H_4_)_3_ under argon in CD_3_OD.  Two C_6_H_4_-F units are disordered over two positions; for one the site occupancy factors are *ca.* 0.53 and 0.47, for the other the values are *ca.* 0.64 and 0.36. The methyl H atoms of the solvent molecule are disordered across the mirror plane.

## Related literature

For related literature, see: Beck *et al.* (1999 ▶) and references therein; Bennett & Donaldson (1977 ▶); Bennett *et al.* (1971 ▶); Evans *et al.* (1999 ▶); Higham *et al.* (2004 ▶); Hoye *et al.* (1993 ▶); Jones *et al.* (1980 ▶); Lorenzini *et al.* (2007*a* ▶,*b* ▶,*c* ▶, 2008*a* ▶,*b* ▶); Montelatici *et al.* (1968 ▶); Young *et al.* (1965 ▶). 
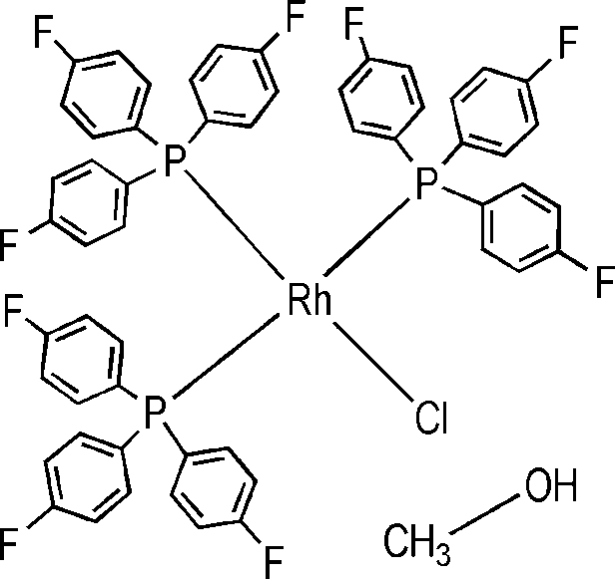


## Experimental

### 

#### Crystal data

[RhCl(C_18_H_12_F_3_P)_3_]·CH_4_O
*M* *_r_* = 1119.14
Monoclinic, 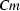

*a* = 10.831 (3) Å
*b* = 23.724 (7) Å
*c* = 9.845 (3) Å
β = 108.213 (8)°
*V* = 2403.0 (12) Å^3^
*Z* = 2
Mo *K*α radiation
μ = 0.59 mm^−1^
*T* = 173.0 (1) K
0.30 × 0.15 × 0.03 mm

#### Data collection

Bruker X8 APEXII diffractometer
Absorption correction: multi-scan (*SADABS*; Bruker, 2003 ▶) *T*_min_ = 0.544, *T*_max_ = 0.983
10921 measured reflections
3312 independent reflections
3094 reflections with *I* > 2σ(*I*)
*R*_int_ = 0.048

#### Refinement

*R*[*F* ^2^ > 2σ(*F* ^2^)] = 0.034
*wR*(*F* ^2^) = 0.075
*S* = 1.03
3312 reflections
372 parameters
15 restraints
H atoms treated by a mixture of independent and constrained refinement
Δρ_max_ = 0.67 e Å^−3^
Δρ_min_ = −0.47 e Å^−3^
Absolute structure: Flack (1983 ▶), 812 Friedel pairs
Flack parameter: −0.03 (3)

### 

Data collection: *SAINT* (Bruker, 2003 ▶); cell refinement: *SAINT*; data reduction: *SAINT*; program(s) used to solve structure: *SIR97* (Altomare *et al.*, 1999 ▶); program(s) used to refine structure: *SHELXL97* (Sheldrick, 2008 ▶); molecular graphics: *ORTEP-3* (Farrugia, 1997 ▶); software used to prepare material for publication: *WinGX* (Farrugia, 1999 ▶).

## Supplementary Material

Crystal structure: contains datablocks I, global. DOI: 10.1107/S1600536808005485/rk2075sup1.cif

Structure factors: contains datablocks I. DOI: 10.1107/S1600536808005485/rk2075Isup2.hkl

Additional supplementary materials:  crystallographic information; 3D view; checkCIF report

## Figures and Tables

**Table 1:** Hydrogen-bond geometry (Å, °)

*D*—H⋯*A*	*D*—H	H⋯*A*	*D*⋯*A*	*D*—H⋯*A*
C3—H3⋯O1^i^	0.95	2.51	3.458 (9)	172
O1—H1*O*⋯Cl1^ii^	1.03 (5)	2.34 (5)	3.369 (9)	174 (11)

Symmetry codes: (i) 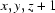; (ii) 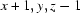.

**Fig. 1.**
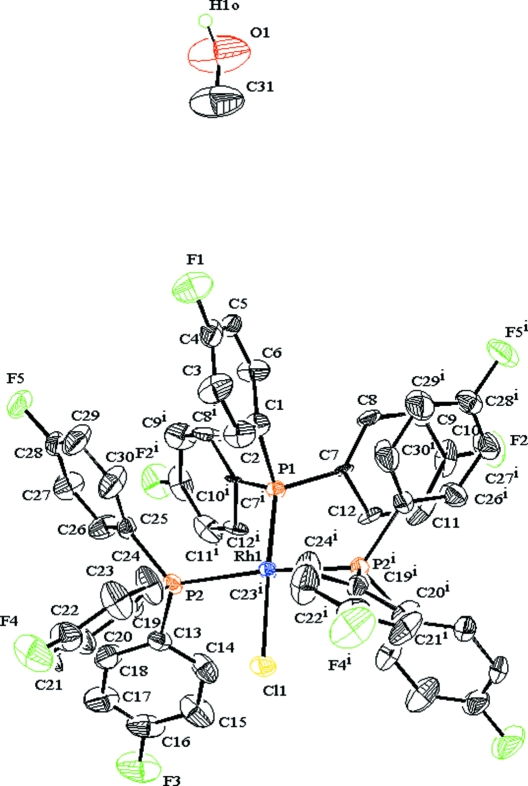
The molecular structure of title compound, with atom labeling scheme. Displacement ellipsoids are drawn at 50% probability level. H atoms are presented as a small spheres of arbitrary radius. Only major part of disordered molecular moieties are presented.

## supplementary crystallographic information

### Comment

We have reported recently on the syntheses of water-soluble Rh^I^–thp complexes such as RhCl(cod)(thp), where thp = tris(hydroxymethyl)phosphine, P(CH_2_OH)_3_, and cod = 1,5-cyclooctadiene (Lorenzini *et al.*, 2007*a*). This complex reacts with P*RR*'_2_ phosphines (*R* = or ≠ *R*') in solution under Ar to generate, concomitantly with *R*'H, the phosphine-phosphinite derivatives RhCl(P*RR*'_2_)[*P*,*P*-*R*'(*R*)POCH_2_P(CH_2_OH)_2_] in two isomeric *cis*- and *trans*-forms, where *cis* and *trans* refer to the disposition of the P atoms with the *R* and *R*' substituents. In some of these systems, trace amounts of the *trans*-RhCl(CO)(P*RR*'_2_)_2_ complexes are formed (Lorenzini *et al.*, 2007*b*), and these have been characterized by X-ray crystallography, for example, for the PEtPh_2_ and *P*(*p*-tolyl)_3_ systems (Lorenzini *et al.*, 2008*b*; Lorenzini *et al.*, submitted). The CO ligand almost certainly derives from the formaldehyde (Beck *et al.*, 1999), which can be readily formed from transition metal–thp species (Higham *et al.*, 2004; Hoye *et al.*, 1993). The RhCl(cod)(thp)/phosphine reactions, when carried out under H_2_, give high yield formation of the dihydrido complexes *cis*,*mer*-Rh(H)_2_Cl(P*RR*'_2_)_3_ (where *R*' = Ph, and *R* = Me or Cy), although in some systems (*e.g.* with PMePh_2_) partial loss of H_2_ occurs and the RhCl(P*RR*'_2_)_3_ species has been detected in solution (Lorenzini *et al.*, 2007*c*). Now, during a reaction of the Rh precursor with P(*p*-FC_6_H_4_)_3_ in MeOH under Ar, we have found that a few crystals of RhCl[P(*p*-FC_6_H_4_)_3_]_3_.CH_3_OH in low overall yield are precipitated.

The so-called "Wilkinson" hydrogenation catalyst, RhCl(PPh_3_)_3_, was first reported in 1965 (Young *et al.*, 1965), and since then 22 Rh(I) complexes of general formula RhCl(P*RR*'_2_)_3_ have been structurally characterized; the first such complex was RhCl(PF_2_NEt_2_)_2_(PPh_3_) (Bennett *et al.*, 1971), while there are just 3 of the type RhCl(P*R*_3_)_3_ where *R* = Ph (Bennett & Donaldson, 1977), Me (Jones *et al.*, 1980) and OPh (Evans *et al.*, 1999). The title P(*p*-FC_6_H_4_)_3_ complex was first isolated in 1968 (Montelatici *et al.*, 1968), but an X-ray structure has not been reported.

### Experimental

General. The RhCl(1,5-cod)(thp) complex was synthesized by our recently reported method (Lorenzini *et al.*, 2007*a*); P(*p*-FC_6_H_4_)_3_ was used as received from Strem Chemicals, CD_3_OD (Cambridge Isotope Laboratory) was used as received. The Rh-phosphine reaction was carried out under Ar using standard Schlenk techniques.

RhCl[P(*p*-FC_6_H_4_)_3_]_3_.CH_3_OH. Addition of P(*p*-FC_6_H_4_)_3_ (10 mg, 0.031 mmol) in CD_3_OD (0.5 ml) to a yellow CD_3_OD solution (0.5 ml) of RhCl(1,5-cod)(thp) (5.6 mg, 0.015 mmol) at room temperature under Ar results in the immediate formation of a brown solution. Over 12 h, a few X-ray quality, yellow plate crystals of the solvated complex deposit from the solution.

### Refinement

The material crystallizes with one molecule of solvent MeOH in the asymmetric unit. Two of the C_6_H_4_F substituents are disordered in two orientations and these were refined with constraints to maintain reasonable geometry and thermal parameters. All non-hydrogen atoms were refined anisotropically, while all hydrogen atoms were placed in calculated positions and not refined, except for H10 which was located in a difference map and refined isotropically.

### Crystal data

**Table:**

[RhCl(C_18_H_12_F_3_P_1_)_3_]·CH_4_O	*F*_000_ = 1132
*M**_r_* = 1119.14	*D*_x_ = 1.547 Mg m^−^^3^
Monoclinic, *C**m*	Mo *K*α radiation λ = 0.71073 Å
*a* = 10.831 (3) Å	Cell parameters from 3285 reflections
*b* = 23.724 (7) Å	θ = 3.3–23.2º
*c* = 9.845 (3) Å	µ = 0.59 mm^−^^1^
β = 108.213 (8)º	*T* = 173.0 (1) K
*V* = 2403.0 (12) Å^3^	Plate, yellow
*Z* = 2	0.30 × 0.15 × 0.03 mm

### Data collection

**Table:**

Bruker X8 APEXII diffractometer	3312 independent reflections
Radiation source: Fine-focus sealed tube	3094 reflections with *I* > 2σ(*I*)
Monochromator: Graphite	*R*_int_ = 0.048
*T* = 173.0(1) K	θ_max_ = 26.3º
Area detector scans	θ_min_ = 1.7º
Absorption correction: multi-scan(SADABS; Bruker, 2003)	*h* = −13→12
*T*_min_ = 0.544, *T*_max_ = 0.983	*k* = −29→29
10921 measured reflections	*l* = −4→12

### Refinement

**Table:**

Refinement on *F*^2^	Hydrogen site location: Geom
Least-squares matrix: Full	H atoms treated by a mixture of independent and constrained refinement
*R*[*F*^2^ > 2σ(*F*^2^)] = 0.034	*w* = 1/[σ^2^(*F*_o_^2^) + (0.0308*P*)^2^ + 3.2966*P*] where *P* = (*F*_o_^2^ + 2*F*_c_^2^)/3
*wR*(*F*^2^) = 0.075	(Δ/σ)_max_ = 0.016
*S* = 1.03	Δρ_max_ = 0.67 e Å^−^^3^
3312 reflections	Δρ_min_ = −0.47 e Å^−^^3^
372 parameters	Extinction correction: None
15 restraints	Absolute structure: Flack (1983), 812 Friedel pairs
Primary atom site location: Direct	Flack parameter: −0.03 (3)
Secondary atom site location: Difmap	

### Special details

**Table:**

Geometry. All s.u.s' (except the s.u. in the dihedral angle between two l.s. planes) are estimated using the full covariance matrix. The cell s.u.s' are taken into account individually in the estimation of s.u.s' in distances, angles and torsion angles; correlations between s.u.s' in cell parameters are only used when they are defined by crystal symmetry. An approximate (isotropic) treatment of cell s.u.s' is used for estimating s.u.s' involving l.s. planes.
Refinement. Refinement of *F*^2^ against ALL reflections. The weighted *R*-factor *wR* and goodness of fit *S* are based on *F*^2^, conventional *R*-factors *R* are based on *F*, with *F* set to zero for negative *F*^2^. The threshold expression of *F*^2^ > 2σ(*F*^2^) is used only for calculating *R*-factors(gt) *etc*. and is not relevant to the choice of reflections for refinement. *R*-factors based on *F*^2^ are statistically about twice as large as those based on *F*, and *R*-factors based on ALL data will be even larger.

### Fractional atomic coordinates and isotropic or equivalent isotropic displacement parameters (Å^2^)

**Table:**

	*x*	*y*	*z*	*U*_iso_*/*U*_eq_	Occ. (<1)
C1	0.2479 (3)	0.0000	0.8201 (5)	0.0317 (16)	
C2	0.3484 (5)	0.0002 (5)	0.9489 (4)	0.0402 (19)	
H2	0.3302	−0.0051	1.0364	0.048*	0.50
C3	0.4758 (4)	0.0083 (6)	0.9494 (6)	0.049 (4)	0.50
H3	0.5444	0.0098	1.0375	0.059*	0.50
C4	0.5028 (4)	0.0142 (4)	0.8210 (8)	0.044 (4)	0.50
C5	0.4023 (6)	0.0127 (4)	0.6922 (6)	0.038 (4)	0.50
H5	0.4207	0.0167	0.6045	0.046*	0.50
C6	0.2748 (5)	0.0053 (4)	0.6917 (4)	0.042 (2)	0.50
H6	0.2062	0.0040	0.6037	0.051*	0.50
C7	0.0049 (17)	−0.0575 (8)	0.721 (2)	0.018 (3)	0.533 (12)
C8	0.0491 (13)	−0.0863 (8)	0.6226 (19)	0.039 (3)	0.533 (12)
H8	0.1295	−0.0761	0.6098	0.047*	0.533 (12)
C9	−0.0245 (12)	−0.1298 (7)	0.5426 (13)	0.048 (4)	0.533 (12)
H9	0.0057	−0.1495	0.4750	0.058*	0.533 (12)
C10	−0.1423 (11)	−0.1447 (5)	0.5614 (13)	0.041 (3)	0.533 (12)
C11	−0.1864 (11)	−0.1159 (6)	0.6603 (16)	0.042 (3)	0.533 (12)
H11	−0.2669	−0.1260	0.6731	0.050*	0.533 (12)
C12	−0.1129 (16)	−0.0723 (8)	0.7403 (19)	0.024 (2)	0.533 (12)
H12	−0.1430	−0.0527	0.8078	0.029*	0.533 (12)
F2	−0.2133 (11)	−0.1847 (4)	0.4740 (9)	0.060 (3)	0.533 (12)
C13	−0.0508 (5)	0.1306 (2)	1.1071 (5)	0.0280 (11)	
C14	−0.1577 (5)	0.0973 (2)	1.0925 (7)	0.0429 (14)	
H14	−0.1535	0.0581	1.0741	0.051*	
C15	−0.2723 (6)	0.1201 (3)	1.1043 (7)	0.0586 (19)	
H15	−0.3464	0.0969	1.0928	0.070*	
C16	−0.2767 (6)	0.1756 (3)	1.1322 (7)	0.0537 (17)	
C17	−0.1751 (8)	0.2093 (3)	1.1419 (10)	0.076 (3)	
H17	−0.1804	0.2486	1.1584	0.091*	
C18	−0.0642 (7)	0.1867 (2)	1.1280 (8)	0.061 (2)	
H18	0.0067	0.2111	1.1330	0.073*	
C19	0.2189 (3)	0.11444 (16)	1.2662 (4)	0.0291 (11)	0.637 (11)
C20	0.2075 (5)	0.1632 (2)	1.3398 (6)	0.037 (2)	0.637 (11)
H20	0.1344	0.1872	1.3032	0.044*	0.637 (11)
C21	0.3031 (5)	0.1768 (2)	1.4668 (6)	0.049 (3)	0.637 (11)
H21	0.2954	0.2101	1.5171	0.058*	0.637 (11)
C22	0.4101 (4)	0.1417 (2)	1.5203 (4)	0.0458 (15)	0.637 (11)
C23	0.4214 (5)	0.0929 (2)	1.4468 (6)	0.054 (3)	0.637 (11)
H23	0.4945	0.0689	1.4833	0.064*	0.637 (11)
C24	0.3258 (5)	0.0793 (2)	1.3197 (6)	0.050 (3)	0.637 (11)
H24	0.3336	0.0460	1.2694	0.060*	0.637 (11)
C25	0.1512 (5)	0.14409 (19)	0.9781 (5)	0.0245 (10)	
C26	0.0727 (5)	0.1841 (2)	0.8909 (6)	0.0413 (13)	
H26	−0.0157	0.1870	0.8876	0.050*	
C27	0.1193 (5)	0.2199 (2)	0.8088 (6)	0.0450 (14)	
H27	0.0638	0.2473	0.7500	0.054*	
C28	0.2439 (5)	0.2159 (2)	0.8121 (6)	0.0354 (12)	
C29	0.3242 (6)	0.1770 (3)	0.8920 (8)	0.0560 (18)	
H29	0.4116	0.1740	0.8918	0.067*	
C30	0.2765 (5)	0.1413 (2)	0.9747 (8)	0.0524 (18)	
H30	0.3330	0.1137	1.0315	0.063*	
F1	0.6241 (6)	0.0176 (3)	0.8185 (10)	0.078 (3)	0.50
F3	−0.3864 (4)	0.19782 (18)	1.1463 (5)	0.0806 (13)	
F4	0.5006 (3)	0.1544 (2)	1.6443 (4)	0.0669 (13)	
F5	0.2899 (3)	0.25244 (13)	0.7328 (4)	0.0531 (9)	
P1	0.08546 (18)	0.0000	0.84167 (19)	0.0197 (4)	
P2	0.09765 (13)	0.09686 (4)	1.09570 (12)	0.0229 (3)	
Cl1	0.05281 (19)	0.0000	1.2983 (2)	0.0321 (5)	
Rh1	0.08365 (4)	0.0000	1.06599 (4)	0.01826 (13)	
O1	0.7332 (8)	0.0000	0.2629 (9)	0.120 (4)	
C31	0.7118 (13)	0.0000	0.3817 (15)	0.104 (5)	
H31A	0.7165	−0.0387	0.4179	0.156*	0.50
H31B	0.6250	0.0154	0.3695	0.156*	0.50
H31C	0.7772	0.0233	0.4500	0.156*	0.50
C7B	−0.0176 (19)	−0.0581 (10)	0.739 (2)	0.018 (3)	0.467 (12)
C8B	0.0088 (14)	−0.0833 (10)	0.623 (2)	0.039 (3)	0.467 (12)
H8B	0.0889	−0.0759	0.6062	0.047*	0.467 (12)
C9B	−0.0818 (13)	−0.1192 (8)	0.5333 (15)	0.048 (4)	0.467 (12)
H9B	−0.0637	−0.1364	0.4545	0.058*	0.467 (12)
C10B	−0.1989 (12)	−0.1299 (6)	0.5585 (15)	0.041 (3)	0.467 (12)
C11B	−0.2254 (14)	−0.1048 (8)	0.6739 (19)	0.042 (3)	0.467 (12)
H11B	−0.3054	−0.1121	0.6912	0.050*	0.467 (12)
C12B	−0.135 (2)	−0.0689 (10)	0.764 (2)	0.024 (2)	0.467 (12)
H12B	−0.1528	−0.0517	0.8430	0.029*	0.467 (12)
F2B	−0.2822 (12)	−0.1659 (5)	0.4806 (11)	0.061 (3)	0.467 (12)
C19B	0.2189 (3)	0.11444 (16)	1.2662 (4)	0.0291 (11)	0.363 (11)
C20B	0.1878 (8)	0.1313 (8)	1.3842 (10)	0.038 (4)	0.363 (11)
H20B	0.0990	0.1368	1.3766	0.046*	0.363 (11)
C21B	0.2825 (8)	0.1408 (8)	1.5153 (10)	0.047 (5)	0.363 (11)
H21B	0.2589	0.1464	1.5995	0.056*	0.363 (11)
C22B	0.4101 (4)	0.1417 (2)	1.5203 (4)	0.0458 (15)	0.363 (11)
C23B	0.4461 (8)	0.1307 (11)	1.4004 (11)	0.067 (7)	0.363 (11)
H23B	0.5349	0.1330	1.4049	0.080*	0.363 (11)
C24B	0.3518 (8)	0.1163 (11)	1.2728 (11)	0.079 (9)	0.363 (11)
H24B	0.3761	0.1077	1.1905	0.095*	0.363 (11)
H1O	0.833 (5)	0.0000	0.282 (14)	0.11 (4)*	

### Atomic displacement parameters (Å^2^)

**Table:**

	*U*^11^	*U*^22^	*U*^33^	*U*^12^	*U*^13^	*U*^23^
C1	0.029 (4)	0.038 (4)	0.026 (4)	0.000	0.006 (3)	0.000
C2	0.028 (4)	0.060 (5)	0.028 (4)	0.000	0.001 (3)	0.000
C3	0.020 (4)	0.074 (12)	0.048 (6)	−0.007 (7)	0.002 (4)	0.004 (8)
C4	0.032 (5)	0.023 (9)	0.083 (9)	0.005 (4)	0.028 (6)	−0.001 (5)
C5	0.043 (6)	0.022 (11)	0.060 (7)	−0.002 (4)	0.033 (6)	0.002 (4)
C6	0.028 (4)	0.068 (7)	0.029 (4)	−0.002 (10)	0.008 (3)	−0.003 (10)
C7	0.024 (6)	0.028 (3)	0.008 (5)	−0.006 (4)	0.011 (4)	0.003 (3)
C8	0.051 (8)	0.047 (4)	0.031 (3)	−0.025 (7)	0.031 (6)	−0.012 (3)
C9	0.065 (11)	0.048 (7)	0.033 (4)	−0.021 (8)	0.016 (7)	−0.021 (4)
C10	0.026 (7)	0.037 (7)	0.034 (4)	−0.011 (5)	−0.028 (6)	0.005 (5)
C11	0.011 (8)	0.059 (7)	0.044 (5)	−0.008 (6)	−0.009 (5)	0.023 (5)
C12	0.017 (6)	0.039 (4)	0.020 (6)	−0.007 (4)	0.009 (4)	0.006 (3)
F2	0.072 (7)	0.051 (5)	0.044 (5)	−0.021 (5)	−0.001 (5)	−0.021 (4)
C13	0.037 (3)	0.026 (3)	0.023 (3)	0.010 (2)	0.013 (2)	0.008 (2)
C14	0.033 (3)	0.042 (3)	0.047 (4)	0.011 (2)	0.002 (3)	−0.015 (3)
C15	0.031 (3)	0.073 (5)	0.065 (5)	0.014 (3)	0.004 (3)	−0.021 (4)
C16	0.059 (4)	0.063 (4)	0.049 (4)	0.040 (3)	0.030 (3)	0.023 (3)
C17	0.116 (6)	0.033 (4)	0.116 (7)	0.039 (4)	0.089 (6)	0.027 (4)
C18	0.084 (5)	0.028 (3)	0.098 (6)	0.011 (3)	0.069 (4)	0.010 (3)
C19	0.037 (3)	0.023 (2)	0.028 (3)	0.002 (2)	0.010 (2)	−0.001 (2)
C20	0.027 (4)	0.040 (5)	0.040 (5)	−0.004 (4)	0.006 (4)	−0.016 (4)
C21	0.045 (5)	0.046 (6)	0.055 (7)	−0.005 (5)	0.015 (5)	−0.040 (5)
C22	0.043 (3)	0.062 (4)	0.029 (3)	−0.005 (3)	0.005 (3)	−0.007 (3)
C23	0.062 (7)	0.046 (6)	0.038 (6)	0.014 (5)	−0.006 (5)	−0.004 (5)
C24	0.057 (6)	0.037 (6)	0.036 (6)	0.010 (5)	−0.012 (5)	−0.010 (5)
C25	0.029 (3)	0.022 (2)	0.021 (2)	−0.002 (2)	0.005 (2)	−0.0011 (19)
C26	0.034 (3)	0.049 (3)	0.043 (3)	0.008 (3)	0.016 (3)	0.019 (3)
C27	0.047 (3)	0.047 (3)	0.038 (3)	0.009 (3)	0.010 (3)	0.020 (3)
C28	0.047 (3)	0.032 (3)	0.030 (3)	−0.014 (2)	0.015 (2)	−0.005 (2)
C29	0.038 (3)	0.045 (4)	0.093 (6)	−0.004 (3)	0.032 (4)	0.014 (4)
C30	0.033 (3)	0.037 (3)	0.090 (6)	0.004 (3)	0.023 (3)	0.020 (3)
F1	0.032 (3)	0.097 (10)	0.119 (7)	−0.008 (3)	0.041 (4)	−0.013 (5)
F3	0.075 (3)	0.090 (3)	0.090 (3)	0.056 (2)	0.043 (2)	0.019 (2)
F4	0.047 (2)	0.094 (3)	0.046 (2)	−0.003 (2)	−0.0053 (18)	−0.032 (2)
F5	0.074 (2)	0.0430 (19)	0.050 (2)	−0.0210 (16)	0.0305 (17)	0.0044 (16)
P1	0.0215 (9)	0.0229 (10)	0.0141 (9)	0.000	0.0044 (7)	0.000
P2	0.0313 (7)	0.0197 (5)	0.0174 (8)	0.0021 (6)	0.0073 (6)	−0.0005 (4)
Cl1	0.0425 (12)	0.0369 (11)	0.0193 (9)	0.000	0.0133 (8)	0.000
Rh1	0.0222 (3)	0.0185 (2)	0.0137 (2)	0.000	0.00499 (19)	0.000
O1	0.072 (6)	0.245 (13)	0.037 (5)	0.000	0.006 (4)	0.000
C31	0.086 (10)	0.177 (16)	0.060 (9)	0.000	0.038 (8)	0.000
C7B	0.024 (6)	0.028 (3)	0.008 (5)	−0.006 (4)	0.011 (4)	0.003 (3)
C8B	0.051 (8)	0.047 (4)	0.031 (3)	−0.025 (7)	0.031 (6)	−0.012 (3)
C9B	0.065 (11)	0.048 (7)	0.033 (4)	−0.021 (8)	0.016 (7)	−0.021 (4)
C10B	0.026 (7)	0.037 (7)	0.034 (4)	−0.011 (5)	−0.028 (6)	0.005 (5)
C11B	0.011 (8)	0.059 (7)	0.044 (5)	−0.008 (6)	−0.009 (5)	0.023 (5)
C12B	0.017 (6)	0.039 (4)	0.020 (6)	−0.007 (4)	0.009 (4)	0.006 (3)
F2B	0.059 (7)	0.054 (7)	0.046 (6)	−0.026 (5)	−0.017 (5)	−0.012 (5)
C19B	0.037 (3)	0.023 (2)	0.028 (3)	0.002 (2)	0.010 (2)	−0.001 (2)
C20B	0.032 (8)	0.055 (12)	0.031 (9)	−0.011 (8)	0.012 (7)	−0.018 (8)
C21B	0.044 (10)	0.057 (13)	0.037 (10)	−0.028 (9)	0.009 (8)	−0.031 (9)
C22B	0.043 (3)	0.062 (4)	0.029 (3)	−0.005 (3)	0.005 (3)	−0.007 (3)
C23B	0.026 (9)	0.13 (2)	0.049 (12)	0.016 (11)	0.014 (8)	0.003 (12)
C24B	0.080 (15)	0.13 (2)	0.015 (8)	0.065 (15)	0.002 (9)	−0.008 (11)

### Geometric parameters (Å, °)

**Table:**

C1—C2	1.3897	C23—H23	0.9500
C1—C6	1.3888	C24—H24	0.9500
C1—P1	1.836 (4)	C25—C30	1.370 (7)
C2—C3	1.3922	C25—C26	1.381 (7)
C2—H2	0.9500	C25—P2	1.831 (5)
C3—C4	1.3900	C26—C27	1.372 (7)
C3—H3	0.9500	C26—H26	0.9500
C4—F1	1.324 (7)	C27—C28	1.343 (7)
C4—C5	1.3900	C27—H27	0.9500
C5—C6	1.3900	C28—C29	1.343 (8)
C5—H5	0.9500	C28—F5	1.360 (6)
C6—H6	0.9500	C29—C30	1.381 (8)
C7—C8	1.3900	C29—H29	0.9500
C7—C12	1.3900	C30—H30	0.9500
C7—P1	1.838 (10)	F1—F1^i^	0.833 (15)
C8—C9	1.3900	F1—C4^i^	1.521 (8)
C8—H8	0.9500	P1—C1^i^	1.836 (4)
C9—C10	1.3900	P1—C7^i^	1.838 (10)
C9—H9	0.9500	P1—C7B	1.862 (13)
C10—F2	1.350 (13)	P1—C7B^i^	1.862 (13)
C10—C11	1.3900	P1—Rh1	2.215 (2)
C11—C12	1.3900	P2—Rh1	2.3153 (12)
C11—H11	0.9500	Cl1—Rh1	2.412 (2)
C12—H12	0.9500	Rh1—P2^i^	2.3153 (12)
C13—C18	1.362 (7)	O1—C31	1.262 (15)
C13—C14	1.372 (7)	O1—H1O	1.07 (4)
C13—P2	1.830 (5)	C31—H31A	0.9800
C14—C15	1.391 (8)	C31—H31B	0.9800
C14—H14	0.9500	C31—H31C	0.9800
C15—C16	1.351 (9)	C7B—C8B	1.3900
C15—H15	0.9500	C7B—C12B	1.3900
C16—C17	1.339 (10)	C8B—C9B	1.3900
C16—F3	1.346 (6)	C8B—H8B	0.9500
C17—C18	1.361 (8)	C9B—C10B	1.3900
C17—H17	0.9500	C9B—H9B	0.9500
C18—H18	0.9500	C10B—F2B	1.303 (15)
C19—C20	1.3900	C10B—C11B	1.3900
C19—C24	1.3900	C11B—C12B	1.3900
C19—P2	1.827 (3)	C11B—H11B	0.9500
C20—C21	1.3900	C12B—H12B	0.9500
C20—H20	0.9500	C20B—C21B	1.393 (8)
C21—C22	1.3900	C20B—H20B	0.9500
C21—H21	0.9500	C21B—H21B	0.9500
C22—F4	1.340 (4)	C23B—C24B	1.392 (8)
C22—C23	1.3900	C23B—H23B	0.9500
C23—C24	1.3900	C24B—H24B	0.9500
			
C2—C1—C6	120.2	C27—C26—C25	121.6 (5)
C2—C1—P1	113.6 (3)	C27—C26—H26	119.2
C6—C1—P1	125.9 (3)	C25—C26—H26	119.2
C1—C2—C3	119.8	C28—C27—C26	119.6 (5)
C1—C2—H2	120.1	C28—C27—H27	120.2
C3—C2—H2	120.1	C26—C27—H27	120.2
C4—C3—C2	120.0	C29—C28—C27	121.6 (5)
C4—C3—H3	120.0	C29—C28—F5	119.2 (5)
C2—C3—H3	120.0	C27—C28—F5	119.1 (5)
F1—C4—C5	118.9 (6)	C28—C29—C30	118.3 (5)
F1—C4—C3	120.9 (6)	C28—C29—H29	120.8
C5—C4—C3	120.0	C30—C29—H29	120.8
C6—C5—C4	120.0	C25—C30—C29	122.7 (5)
C6—C5—H5	120.0	C25—C30—H30	118.7
C4—C5—H5	120.0	C29—C30—H30	118.7
C5—C6—C1	120.0	C1^i^—P1—C7	101.8 (5)
C5—C6—H6	120.0	C1—P1—C7	101.8 (5)
C1—C6—H6	120.0	C1^i^—P1—C7^i^	101.8 (5)
C8—C7—C12	120.0	C1—P1—C7^i^	101.8 (5)
C8—C7—P1	128.1 (9)	C7—P1—C7^i^	95.9 (17)
C12—C7—P1	111.9 (9)	C1^i^—P1—C7B	111.7 (6)
C7—C8—C9	120.0	C1—P1—C7B	111.7 (6)
C7—C8—H8	120.0	C7^i^—P1—C7B	96.7 (3)
C9—C8—H8	120.0	C1^i^—P1—C7B^i^	111.7 (6)
C10—C9—C8	120.0	C1—P1—C7B^i^	111.7 (6)
C10—C9—H9	120.0	C7—P1—C7B^i^	96.7 (3)
C8—C9—H9	120.0	C7B—P1—C7B^i^	95.6 (19)
F2—C10—C9	117.2 (9)	C1^i^—P1—Rh1	115.00 (17)
F2—C10—C11	122.7 (9)	C1—P1—Rh1	115.00 (17)
C9—C10—C11	120.0	C7—P1—Rh1	119.4 (7)
C12—C11—C10	120.0	C7^i^—P1—Rh1	119.4 (7)
C12—C11—H11	120.0	C7B—P1—Rh1	110.6 (8)
C10—C11—H11	120.0	C7B^i^—P1—Rh1	110.6 (8)
C11—C12—C7	120.0	C19—P2—C13	103.7 (2)
C11—C12—H12	120.0	C19—P2—C25	99.3 (2)
C7—C12—H12	120.0	C13—P2—C25	103.3 (2)
C18—C13—C14	116.9 (5)	C19—P2—Rh1	110.04 (13)
C18—C13—P2	125.0 (4)	C13—P2—Rh1	114.65 (18)
C14—C13—P2	118.1 (4)	C25—P2—Rh1	123.28 (16)
C13—C14—C15	120.9 (6)	P1—Rh1—P2	96.05 (3)
C13—C14—H14	119.5	P1—Rh1—P2^i^	96.05 (3)
C15—C14—H14	119.5	P2—Rh1—P2^i^	165.90 (5)
C16—C15—C14	119.0 (6)	P1—Rh1—Cl1	172.92 (8)
C16—C15—H15	120.5	P2—Rh1—Cl1	84.45 (3)
C14—C15—H15	120.5	P2^i^—Rh1—Cl1	84.45 (3)
C17—C16—F3	119.4 (6)	C31—O1—H1O	103 (5)
C17—C16—C15	121.1 (5)	O1—C31—H31A	109.5
F3—C16—C15	119.4 (6)	O1—C31—H31B	109.5
C16—C17—C18	119.2 (6)	H31A—C31—H31B	109.5
C16—C17—H17	120.4	O1—C31—H31C	109.5
C18—C17—H17	120.4	H31A—C31—H31C	109.5
C17—C18—C13	122.7 (6)	H31B—C31—H31C	109.5
C17—C18—H18	118.7	C8B—C7B—C12B	120.0
C13—C18—H18	118.7	C8B—C7B—P1	121.4 (11)
C20—C19—C24	120.0	C12B—C7B—P1	117.7 (10)
C20—C19—P2	120.9 (2)	C7B—C8B—C9B	120.0
C24—C19—P2	119.0 (2)	C7B—C8B—H8B	120.0
C19—C20—C21	120.0	C9B—C8B—H8B	120.0
C19—C20—H20	120.0	C10B—C9B—C8B	120.0
C21—C20—H20	120.0	C10B—C9B—H9B	120.0
C20—C21—C22	120.0	C8B—C9B—H9B	120.0
C20—C21—H21	120.0	F2B—C10B—C11B	118.7 (11)
C22—C21—H21	120.0	F2B—C10B—C9B	121.2 (11)
F4—C22—C23	120.2 (4)	C11B—C10B—C9B	120.0
F4—C22—C21	119.8 (4)	C10B—C11B—C12B	120.0
C23—C22—C21	120.0	C10B—C11B—H11B	120.0
C24—C23—C22	120.0	C12B—C11B—H11B	120.0
C24—C23—H23	120.0	C11B—C12B—C7B	120.0
C22—C23—H23	120.0	C11B—C12B—H12B	120.0
C23—C24—C19	120.0	C7B—C12B—H12B	120.0
C23—C24—H24	120.0	C21B—C20B—H20B	119.0
C19—C24—H24	120.0	C20B—C21B—H21B	120.7
C30—C25—C26	116.1 (5)	C24B—C23B—H23B	120.2
C30—C25—P2	119.9 (4)	C23B—C24B—H24B	120.1
C26—C25—P2	124.0 (4)		
			
C6—C1—C2—C3	2.8	C8—C7—P1—C7^i^	93.6 (12)
P1—C1—C2—C3	−170.91 (16)	C12—C7—P1—C7^i^	−87.8 (10)
C1—C2—C3—C4	−2.2	C12—C7—P1—C7B	7(9)
C2—C3—C4—F1	−174.8 (7)	C8—C7—P1—C7B^i^	104.1 (13)
C2—C3—C4—C5	0.8	C12—C7—P1—C7B^i^	−77.3 (10)
F1—C4—C5—C6	175.7 (7)	C8—C7—P1—Rh1	−137.7 (10)
C3—C4—C5—C6	0.0	C12—C7—P1—Rh1	40.9 (11)
C4—C5—C6—C1	0.6	C20—C19—P2—C13	−27.6 (5)
C2—C1—C6—C5	−2.0	C24—C19—P2—C13	154.7 (4)
P1—C1—C6—C5	170.89 (19)	C20—C19—P2—C25	78.7 (5)
C12—C7—C8—C9	0.0	C24—C19—P2—C25	−99.1 (4)
P1—C7—C8—C9	178.5 (18)	C20—C19—P2—Rh1	−150.6 (4)
C7—C8—C9—C10	0.0	C24—C19—P2—Rh1	31.6 (4)
C8—C9—C10—F2	175.7 (11)	C18—C13—P2—C19	58.3 (6)
C8—C9—C10—C11	0.0	C14—C13—P2—C19	−122.2 (4)
F2—C10—C11—C12	−175.5 (11)	C18—C13—P2—C25	−44.9 (6)
C9—C10—C11—C12	0.0	C14—C13—P2—C25	134.5 (4)
C10—C11—C12—C7	0.0	C18—C13—P2—Rh1	178.3 (5)
C8—C7—C12—C11	0.0	C14—C13—P2—Rh1	−2.2 (5)
P1—C7—C12—C11	−178.8 (15)	C30—C25—P2—C19	55.9 (5)
C18—C13—C14—C15	−2.4 (9)	C26—C25—P2—C19	−123.3 (5)
P2—C13—C14—C15	178.1 (5)	C30—C25—P2—C13	162.5 (5)
C13—C14—C15—C16	−0.8 (10)	C26—C25—P2—C13	−16.7 (5)
C14—C15—C16—C17	3.1 (11)	C30—C25—P2—Rh1	−65.7 (5)
C14—C15—C16—F3	−178.8 (6)	C26—C25—P2—Rh1	115.1 (4)
F3—C16—C17—C18	179.8 (7)	C1^i^—P1—Rh1—P2	86.36 (4)
C15—C16—C17—C18	−2.1 (12)	C1—P1—Rh1—P2	86.36 (4)
C16—C17—C18—C13	−1.4 (13)	C7—P1—Rh1—P2	−152.1 (8)
C14—C13—C18—C17	3.5 (11)	C7^i^—P1—Rh1—P2	−35.2 (8)
P2—C13—C18—C17	−177.0 (6)	C7B—P1—Rh1—P2	−145.9 (9)
C24—C19—C20—C21	0.0	C7B^i^—P1—Rh1—P2	−41.3 (9)
P2—C19—C20—C21	−177.8 (3)	C1^i^—P1—Rh1—P2^i^	−86.36 (4)
C19—C20—C21—C22	0.0	C1—P1—Rh1—P2^i^	−86.36 (4)
C20—C21—C22—F4	−178.6 (5)	C7—P1—Rh1—P2^i^	35.2 (8)
C20—C21—C22—C23	0.0	C7^i^—P1—Rh1—P2^i^	152.1 (8)
F4—C22—C23—C24	178.6 (5)	C7B—P1—Rh1—P2^i^	41.3 (9)
C21—C22—C23—C24	0.0	C7B^i^—P1—Rh1—P2^i^	145.9 (9)
C22—C23—C24—C19	0.0	C19—P2—Rh1—P1	−134.29 (16)
C20—C19—C24—C23	0.0	C13—P2—Rh1—P1	109.30 (18)
P2—C19—C24—C23	177.8 (3)	C25—P2—Rh1—P1	−17.8 (2)
C30—C25—C26—C27	−1.7 (9)	C19—P2—Rh1—P2^i^	14.6 (4)
P2—C25—C26—C27	177.5 (5)	C13—P2—Rh1—P2^i^	−101.8 (3)
C25—C26—C27—C28	0.4 (10)	C25—P2—Rh1—P2^i^	131.1 (3)
C26—C27—C28—C29	1.3 (10)	C19—P2—Rh1—Cl1	52.81 (16)
C26—C27—C28—F5	−178.5 (5)	C13—P2—Rh1—Cl1	−63.59 (18)
C27—C28—C29—C30	−1.5 (10)	C25—P2—Rh1—Cl1	169.3 (2)
F5—C28—C29—C30	178.3 (6)	C1^i^—P1—C7B—C8B	−21.7 (15)
C26—C25—C30—C29	1.5 (10)	C1—P1—C7B—C8B	−21.7 (15)
P2—C25—C30—C29	−177.7 (6)	C7—P1—C7B—C8B	−2(9)
C28—C29—C30—C25	0.0 (11)	C7^i^—P1—C7B—C8B	83.8 (14)
C5—C4—F1—F1^i^	−90.3 (7)	C7B^i^—P1—C7B—C8B	94.3 (12)
C3—C4—F1—F1^i^	85.4 (5)	Rh1—P1—C7B—C8B	−151.2 (10)
C5—C4—F1—C4^i^	−90.3 (3)	C1^i^—P1—C7B—C12B	169.5 (8)
C3—C4—F1—C4^i^	85.4 (4)	C1—P1—C7B—C12B	169.5 (8)
C2—C1—P1—C7	−130.9 (10)	C7—P1—C7B—C12B	−171 (10)
C6—C1—P1—C7	55.8 (10)	C7^i^—P1—C7B—C12B	−84.9 (10)
C2—C1—P1—C7^i^	130.4 (10)	C7B^i^—P1—C7B—C12B	−74.4 (14)
C6—C1—P1—C7^i^	−42.9 (10)	Rh1—P1—C7B—C12B	40.1 (13)
C2—C1—P1—C7B	−127.4 (11)	C12B—C7B—C8B—C9B	0.0
C6—C1—P1—C7B	59.3 (11)	P1—C7B—C8B—C9B	−168.4 (19)
C2—C1—P1—C7B^i^	126.9 (11)	C7B—C8B—C9B—C10B	0.0
C6—C1—P1—C7B^i^	−46.4 (11)	C8B—C9B—C10B—F2B	−176.0 (14)
C2—C1—P1—Rh1	−0.2 (5)	C8B—C9B—C10B—C11B	0.0
C6—C1—P1—Rh1	−173.6 (5)	F2B—C10B—C11B—C12B	176.1 (13)
C8—C7—P1—C1^i^	−9.8 (14)	C9B—C10B—C11B—C12B	0.0
C12—C7—P1—C1^i^	168.8 (7)	C10B—C11B—C12B—C7B	0.0
C8—C7—P1—C1	−9.8 (14)	C8B—C7B—C12B—C11B	0.0
C12—C7—P1—C1	168.8 (7)	P1—C7B—C12B—C11B	168.9 (19)

Symmetry codes: (i) *x*, −*y*, *z*.

### Hydrogen-bond geometry (Å, °)

**Table:**

*D*—H···*A*	*D*—H	H···*A*	*D*···*A*	*D*—H···*A*
C3—H3···O1^ii^	0.95	2.51	3.458 (9)	172
O1—H1O···Cl1^iii^	1.03 (5)	2.34 (5)	3.369 (9)	174 (11)

Symmetry codes: (ii) *x*, *y*, *z*+1; (iii) *x*+1, *y*, *z*−1.

## References

[bb1] Altomare, A., Burla, M. C., Camalli, M., Cascarano, G. L., Giacovazzo, C., Guagliardi, A., Moliterni, A. G. G., Polidori, G. & Spagna, R. (1999). *J. Appl. Cryst.* **32**, 115–119.

[bb2] Beck, C. M., Rathmill, S. E., Park, Y. J., Chen, J., Crabtree, R. H., Liable-Sands, L. M. & Rheingold, A. L. (1999). *Organometallics*, **18**, 5311–5317.

[bb3] Bennett, M. J. & Donaldson, P. B. (1977). *Inorg. Chem.* **16**, 655–660.

[bb4] Bennett, M. A., Robertson, G. B., Turney, T. W. & Whimp, P. O. (1971). *J. Chem. Soc. D*, pp. 762–764.

[bb5] Bruker (2003). *SADABS.* and *SAINT* Bruker AXS Inc., Madison, Wisconsin, USA.

[bb7] Evans, P. A., Incarvito, C. D. & Rheingold, A. L. (1999). Private communication (deposition number: 115178). CCDC, Cambridge, England.

[bb8] Farrugia, L. J. (1997). *J. Appl. Cryst.* **30**, 565.

[bb9] Farrugia, L. J. (1999). *J. Appl. Cryst.* **32**, 837–838.

[bb10] Flack, H. D. (1983). *Acta Cryst.* A**39**, 876–881.

[bb11] Higham, L. J., Whittlesey, M. K. & Wood, P. T. (2004). *J. Chem. Soc. Dalton Trans.* pp. 4202–4208. 10.1039/b411701h 15573173

[bb12] Hoye, P. A. T., Pringle, P. G., Smith, M. B. & Worboys, K. (1993). *J. Chem. Soc. Dalton Trans.* pp. 269–274.

[bb13] Jones, R. A., Real, F. M., Wilkinson, G., Galas, A. M. R., Hursthouse, M. B. & Malik, K. M. A. (1980). *J. Chem. Soc. Dalton Trans.* pp. 511–518.

[bb14] Lorenzini, F., Patrick, B. O. & James, B. R. (2007*a*). *J. Chem. Soc. Dalton Trans.* pp. 3224–3226. 10.1039/b706691k 17893766

[bb15] Lorenzini, F., Patrick, B. O. & James, B. R. (2007*b*). *Inorg. Chem.* **46**, 8998–9002. 10.1021/ic7012182 17867681

[bb16] Lorenzini, F., Patrick, B. O. & James, B. R. (2007*c*). *Inorg. Chim. Acta*, doi:10.1016/j.ica.2007.10.044.

[bb17] Lorenzini, F., Patrick, B. O. & James, B. R. (2008*a*). *Acta Cryst.* E**64**, m179–m180. 10.1107/S1600536807065877 PMC2915114 21200527

[bb18] Lorenzini, F., Patrick, B. O. & James, B. R. (2008*b*). *Acta Cryst.* E**64**, m464–m465. 10.1107/S1600536808003528 PMC2960764 21201856

[bb19] Montelatici, S., van der Ent, A., Osborn, J. A. & Wilkinson, G. (1968). *J. Chem. Soc. A*, pp. 1054–1058.

[bb20] Sheldrick, G. M. (2008). *Acta Cryst.* A**64**, 112–122. 10.1107/S0108767307043930 18156677

[bb21] Young, J. F., Osborn, J. A., Jardine, F. H. & Wilkinson, G. (1965). *Chem. Commun.* pp. 131–132.

